# An Association Between Posterior Reversible Encephalopathy Syndrome and Severe Hypercapnia in Chronic CO₂ Retention

**DOI:** 10.7759/cureus.98256

**Published:** 2025-12-01

**Authors:** Cameron Yen, Linda Chun, Jay Mahajan, Aroucha Vickers

**Affiliations:** 1 Neurology, Valley Hospital Medical Center, Las Vegas, USA; 2 Neurology, Las Vegas Neurology Center, Las Vegas, USA

**Keywords:** acute hypercapnia, acute hypercapnic respiratory failure, copd (chronic obstructive pulmonary disease), hypercapnia, posterior reversible encephalopathy syndrome (pres), severe hypercapnia, vasogenic brain edema

## Abstract

We discuss a 60-year-old male with a history of chronic obstructive pulmonary disease (COPD) on home oxygen and chronic carbon dioxide (CO₂) retention who initially presented with hypoxemic and hypercapnic respiratory failure and subsequent development of acute encephalopathy. Magnetic resonance imaging (MRI) of the brain showed vasogenic edema in the bilateral occipital lobes. Management with increasing supplemental oxygen and nightly bilevel positive airway pressure (BiPAP) led to rapid neurological improvement. A repeat brain MRI of the brain six days later showed improving edema in the bilateral occipital lobes. This case highlights acute and severe hypercapnia as a potentially uncommon and under-recognized trigger of PRES. It further underscores that prompt correction of hypercapnia can rapidly improve both clinical and radiographic manifestations of PRES.

## Introduction

First described by Hinchey et al. in 1996, PRES is a clinicoradiological disorder characterized by the abrupt onset of neurological symptoms and distinctive neuroimaging findings [1]. It presents with nonspecific but characteristic symptoms, including headache, seizures, encephalopathy, or visual disturbances. MRI of the brain most often demonstrates reversible vasogenic edema involving the posterior circulation, specifically the subcortical white matter of the parieto-occipital regions [2]. Although acute hypertension, renal disease, autoimmune disorders, and exposure to immunosuppressive or cytotoxic agents are well-established risk factors, PRES can also arise from other systemic insults [3,4]. Hypercapnia, defined as an arterial partial pressure of carbon dioxide (PaCO₂) > 45 mm Hg, can alter cerebral blood flow by inducing vasodilation and impairing autoregulatory capacity, potentially increasing susceptibility to vasogenic edema [5,6]. Case reports linking severe hypercapnia to PRES remain limited, but the pathophysiologic plausibility is supported by its effects on cerebral hemodynamics [5,6]. We report a case of PRES occurring in a patient with COPD and chronic CO₂ retention, with acute elevations in PaCO₂ as a potential precipitating factor.

## Case presentation

A 60-year-old male with a history of COPD on 2 liters of home oxygen, chronic CO₂ retention, recurrent aspiration pneumonia, and chronic tobacco use presented with hypoxemic and hypercapnic respiratory failure. At the time of admission, the patient was fully oriented to person, place, time, and situation and able to follow commands with no focal neurological deficits. Arterial blood gas (ABG) revealed a pH of 7.43, PaCO₂ of 75.8 mm Hg, PaO₂ of 27.7 mm Hg, and bicarbonate of 37.2, consistent with chronic compensated respiratory acidosis. The initially reported PaO₂ was likely a sampling error, as it was incompatible with the patient’s clinical status. Nevertheless, a repeat ABG performed shortly thereafter showed a pH of 7.43, a PaCO₂ of 76.1 mm Hg, and a PaO₂ of 58.5 mm Hg, confirming profound hypercapnia. A review of two prior admissions demonstrated initial ABG values also consistent with chronic compensated respiratory acidosis. Chest X-ray demonstrated bilateral pleural effusions, and contrast-enhanced computed tomography (CT) angiography of the chest showed bilateral consolidations with associated effusions. He was empirically started on azithromycin, cefepime, and vancomycin for presumed aspiration pneumonia. His respiratory pathogen panel was unremarkable, and the sputum culture was growing upper respiratory flora. High-flow nasal cannula with a fraction of inspired oxygen (FiO₂) of 60% and breathing treatments with ipratropium-albuterol were started on the day of admission, resulting in improvement in respiratory status. He was transitioned to nasal cannula oxygen at 3 liters with an FiO₂ of 32%. 

**Figure 1:**
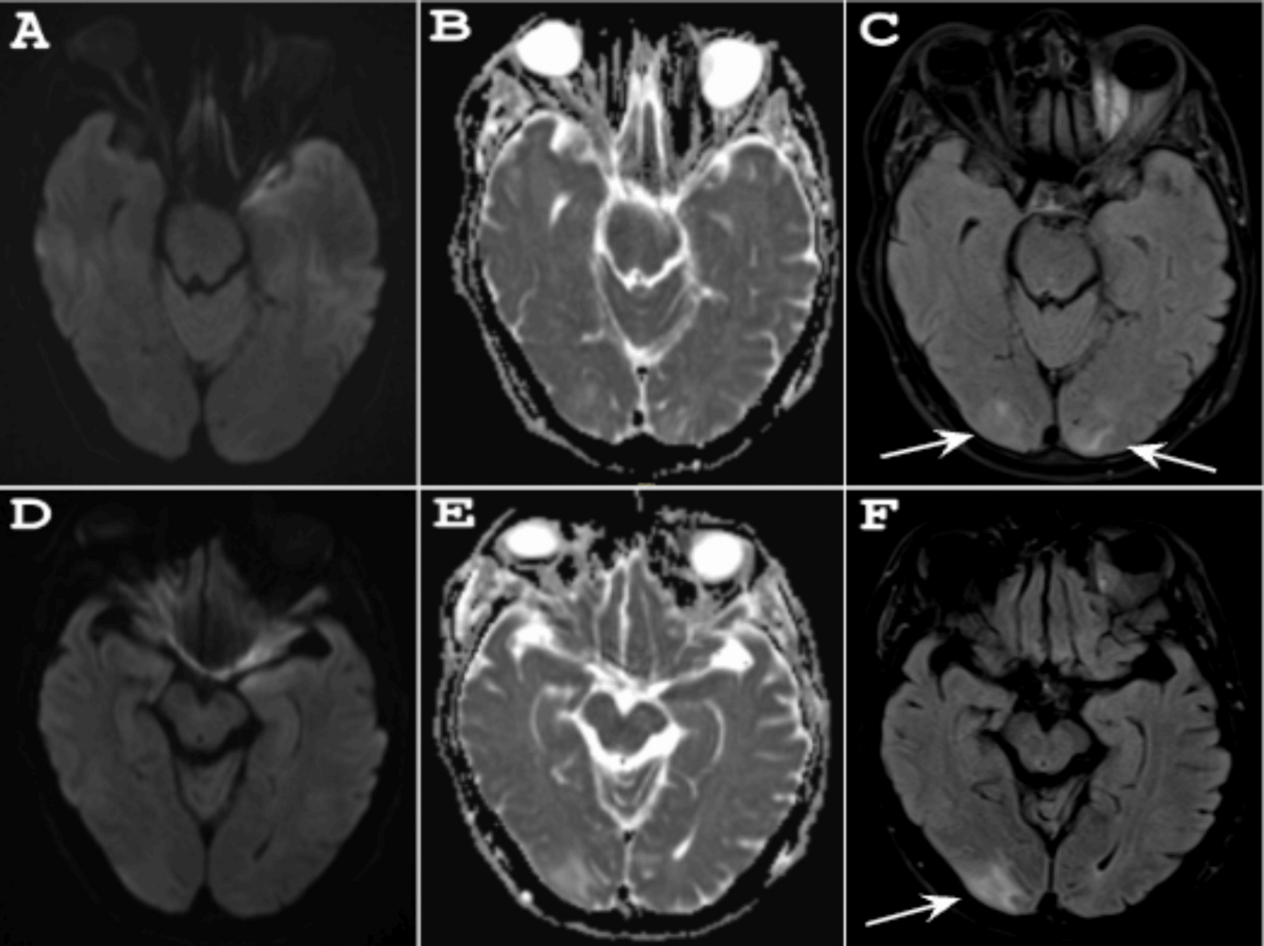
MRI of the brain pre-treatment (A, B, D, E) Axial DWI and ADC showing no evidence of diffusion restriction, while (C, F) FLAIR demonstrates hyperintensities of the right parietal and bilateral occipital lobes suggestive of vasogenic edema. MRI: magnetic resonance imaging; DWI: diffusion-weighted imaging; ADC: apparent diffusion coefficient; FLAIR: fluid-attenuated inversion recovery

Over the next several days, the patient required increasing oxygen supplementation and refused nightly BiPAP therapy because he stated it was too uncomfortable to wear. Subsequently, he became more lethargic. ABG at this time revealed a pH of 7.22, PaCO₂ > 102.0 mm Hg, and a normalized PaO₂. The non-contrast CT head was normal. MRI of the brain demonstrated white matter edema in the right parietal and bilateral occipital lobes, suggestive of PRES (Figure 1). 

**Figure 2:**
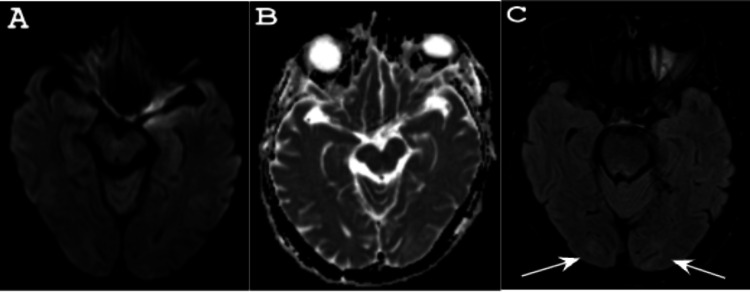
MRI of the brain post-treatment (A, B) Axial DWI and ADC again showing no evidence of diffusion restriction, while (C) FLAIR shows improvement of the bilateral hyperintensities in the right parietal and bilateral occipital lobes following improvement in PaCO₂ levels after six days. MRI: magnetic resonance imaging; DWI: diffusion-weighted imaging; ADC: apparent diffusion coefficient; FLAIR: fluid-attenuated inversion recovery; PaCO₂: arterial partial pressure of carbon dioxide

Neurology was consulted at this time. On initial evaluation, the patient was lethargic, oriented only to name, and required persistent tactile stimulation to arouse him. Attention was markedly impaired with difficulty following most commands due to his somnolence. He was able to move all of his extremities spontaneously with no appreciable focal neurological deficits given the limitations of the exam. Thoracentesis of the pleural effusion was performed, yielding fluid consistent with an exudative process and cultures demonstrating no growth. Given the patient’s encephalopathy, nocturnal BiPAP was initiated after obtaining consent from the patient’s spouse. As PaCO₂ levels improved, the patient’s mental status returned to baseline. A repeat MRI of the brain performed six days later showed improvement of the bilateral hyperintensities in the right parietal and bilateral occipital lobes (Figure 2).

During hospitalization, the patient remained relatively normotensive, with systolic blood pressures ranging from 100 to 140 mm Hg and diastolic pressures from 65 to 90 mm Hg. He ultimately left against medical advice three weeks after admission and was lost to follow-up.

## Discussion

Multiple pathophysiologic mechanisms have been implicated in the development of PRES. The earliest theory suggested that rising blood pressure triggers compensatory autoregulatory vasoconstriction, which reduces cerebral perfusion and induces ischemic stress [7]. This ischemia disrupts the blood-brain barrier, leading to leakage of plasma and proteins into the interstitium and the development of vasogenic edema [8]. The current and more widely cited vasogenic theory suggests that excessive blood pressure leads to forced vasodilation and hyperperfusion, which stresses the capillary endothelium, disrupts the blood-brain barrier, and allows plasma leakage [1]. The posterior cerebral circulation is particularly vulnerable to these hemodynamic shifts due to its relative lack of sympathetic innervation, which impairs its ability to constrict during abrupt rises in blood pressure [4,9]. 

Although the vasogenic theory accounts for many cases, subsequent studies have shown that PRES develops in up to 30% of patients without severe hypertension [10]. These findings prompted alternative explanations centered on endothelial dysfunction as a key factor in the development of PRES. The cytotoxic theory attributes this to direct endothelial injury from endogenous mediators such as chemokines or exogenous agents including chemotherapy and immunosuppressants [11,12]. The immunogenic theory emphasizes an inflammatory trigger, with T-cell activation and cytokine release leading to vascular injury [11,13]. The neuropeptide theory involves the release of vasoactive substances such as endothelin-1 (ET-1), prostacyclin, and thromboxane A2 [11,13]. Each of these theories proposes a different pathway that ultimately leads to cerebral vasoconstriction, ischemia, and the vasogenic edema characteristic of PRES [11,13]. Importantly, they provide a framework for understanding how PRES can develop even in patients who are normotensive or only mildly hypertensive. Given the variability in PRES pathophysiology and etiology, these mechanisms may act in different combinations, and outcomes are largely determined by the underlying cause [4]. Prompt correction of the underlying insult not only improves functional outcomes but also supports the clinical and radiographic reversibility characteristic of PRES [1].

Our case suggests that severe hypercapnia may be an under-recognized precipitant of PRES. While the precise pathophysiologic link between hypercapnia and PRES remains incompletely understood, emerging evidence suggests a multifactorial process involving cerebral endothelial dysfunction and impaired autoregulatory capacity [9,14]. This is consistent with observations that higher PaCO₂ levels correlate with reduced autoregulatory efficiency, as hypercapnia induces cerebral vasodilation, elevates perfusion pressure and flow, and weakens autoregulatory mechanisms [5]. When cerebral autoregulation fails, regions of hyperperfusion can develop, leading to capillary leakage and vasogenic edema [9]. 

It should also be acknowledged that the patient’s long-standing COPD likely promoted vascular endothelial injury and compromised autoregulatory capacity [14]. COPD involves systemic inflammation marked by peripheral T-cell activation and elevated levels of pro-inflammatory cytokines such as interleukin-1 (IL-1) and tumor necrosis factor-alpha (TNF-α) [14]. This chronic inflammatory state contributes to higher baseline levels of ET-1, which can impair endothelial function in cerebral arteries and blunt the normal vasodilatory response to hypercapnia [15]. Furthermore, COPD is one of the most common pre-existing diseases preceding the onset of hypercapnic respiratory failure [16]. In our patient, his aspiration pneumonia and pleural effusions likely led him to have an acute COPD exacerbation resulting in severe hypercapnia far from his baseline. Although pneumonia has rarely been cited in the development of PRES, it is most often reported in association with sepsis, shock, or COVID-19 [12,17]. In contrast, our patient had none of these systemic complications. Moreover, prior hospitalizations for similar complaints revealed much less pronounced elevations in PaCO₂ than that observed during this admission. Therefore, it may be postulated that acute, severe rises in PaCO₂ are more disruptive than chronic elevations, as chronic hypercapnia can lead to partial vascular adaptation, whereas acute decompensation may exceed compensatory mechanisms and precipitate endothelial failure [18,19]. 

Taken together, these mechanisms and predisposing factors suggest a plausible link between severe hypercapnia and the development of PRES, as illustrated by our case. We propose that the patient’s COPD and chronic hypercapnia conferred a baseline susceptibility to cerebral endothelial dysfunction and that the acute and marked rise in PaCO₂ far from baseline levels was the catalyst in the observed clinical and radiographic manifestations of PRES. Lending further support, prompt correction of PaCO₂ to his baseline levels resulted in clinical and radiographic improvement. This case underscores the importance of recognizing severe hypercapnia as a potential trigger of PRES and adds to the growing understanding of the diverse mechanisms underlying this syndrome.

## Conclusions

In our patient with chronic CO₂ retention due to COPD, acute elevations in PaCO₂ were temporally associated with encephalopathy and characteristic neuroimaging findings of PRES. Rapid improvement in both clinical status and radiographic abnormalities following correction of his severe hypercapnia underscores the reversibility of PRES when the underlying trigger is promptly addressed. Given the growing recognition that PRES may arise from diverse systemic insults beyond hypertension, clinicians should maintain a high index of suspicion for this disorder in patients with profound hypercapnia. As COPD and chronic CO₂ retention are commonly encountered in clinical practice, severe hypercapnia may represent a more under-recognized risk factor for PRES than previously appreciated. Further investigation into the pathophysiologic link between disordered CO₂ homeostasis, cerebral autoregulatory dysfunction, and endothelial injury is warranted to expand our understanding of PRES and to refine strategies for its prevention and management.
